# Non-linear associations of atherogenic index of plasma with prediabetes and type 2 diabetes mellitus among Chinese adults aged 45 years and above: a cross-sectional study from CHARLS

**DOI:** 10.3389/fendo.2024.1360874

**Published:** 2024-04-02

**Authors:** Luqing Jiang, Lei Li, Zichen Xu, Yu Tang, Ying Zhai, Xia Fu, Daoqin Liu, Qiwen Wu

**Affiliations:** ^1^ Department of Laboratory Medicine, the First Affiliated Hospital of Wannan Medical College, Wuhu, Anhui, China; ^2^ Department of Kidney Medicine, the First Affiliated Hospital of Wannan Medical College, Wuhu, Anhui, China

**Keywords:** atherogenic index of plasma, prediabetes, type 2 diabetes mellitus, CHARLS, cross-sectional study

## Abstract

**Background:**

Dyslipidemia is strongly associated with the development of prediabetes and type 2 diabetes mellitus (T2DM). The atherogenic index of plasma (AIP), as a comprehensive index for assessing lipid metabolism, has received extensive attention from researchers in recent years. However, there are relatively few studies exploring the relationships between AIP and the risk of prediabetes and T2DM in the Chinese population. This study focuses on exploring the relationships of AIP with the risk of prediabetes and T2DM in the Chinese population.

**Methods:**

We conducted an analysis of the public data from the China Health and Retirement Longitudinal Study (CHARLS), involving a total of 12,060 participants aged 45 years and above in China. The study explored the relationships of AIP with prediabetes and T2DM risk through multivariate logistic regression, subgroup analysis, smooth curve fitting, and threshold effect analysis.

**Results:**

After adjusting for potential confounding factors, we observed positive associations between AIP and the risk of prediabetes [odds ratio (OR) = 1.75, 95% confidence interval (CI): 1.49–2.06] and T2DM (OR = 2.91, 95% CI: 2.38–3.57). Participants with higher AIP levels demonstrated a significantly elevated risk of prediabetes (OR = 1.52, 95% CI: 1.33–1.74) and T2DM (OR = 2.28, 95% CI: 1.92–2.71) compared to those with lower AIP levels. AIP showed consistent correlations with prediabetes and T2DM risk in different subgroups. The results showed the non-linear relationships between AIP and risk of prediabetes and T2DM, with inflection points at 0.29 and −0.04, respectively. When AIP > 0.29, there was a positive association between AIP and the risk of prediabetes (OR = 2.24, 95% CI: 1.67–3.00, *p* < 0.0001). Similarly, when AIP > −0.04, AIP was positively associated with the risk of T2DM (OR = 3.33, 95% CI: 2.67–4.16, *p* < 0.0001).

**Conclusions:**

This study demonstrated non-linear positive associations of AIP with the risk of prediabetes and T2DM among participants ≥ 45 years of age in China.

## Introduction

Type 2 diabetes mellitus (T2DM) is characterized by impaired pancreatic β-cell function and relative insulin deficiency caused by insulin resistance (1). As one of the most prevalent chronic diseases globally, T2DM has emerged as a significant public health challenge affecting human health. In recent years, the prevalence of T2DM has shown a consistent upward trend in both developed and developing countries (2–4), posing substantial burdens on public health and healthcare systems (5, 6). According to data from the International Diabetes Federation (IDF), the global prevalence of diabetes in the 20–79 age group was estimated to be 10.5% (536.6 million people) in, 2021, with an estimated increase to 12.2% (783.2 million people) by, 2045. In addition, the statistics states that China has the largest number of people with diabetes, with approximately more than 140 million in, 2021 and an estimated more than 174 million by, 2045 (7).

However, there are still a considerable number of individuals with prediabetes. Globally, an estimated 7.5% (374 million people) of adults have prediabetes. Without effective preventive measures, this percentage is expected to reach 8.0% (454 million people) by, 2030 and 8.6% (548 million people) by, 2045 (8). During the prediabetes stage, abnormalities in glucose metabolism begin to emerge, usually accompanied by insulin resistance and dyslipidemia (9). If timely intervention is not made, the risk of progressing to diabetes is significantly elevated (10, 11). However, the majority of individuals with prediabetes remain undiagnosed (12). Accurately estimating prediabetes to identify high-risk individuals is a challenging task. Emphasizing the diagnosis of prediabetes and early intervention are crucial for preventing or delaying the incidence of T2DM and its complications.

Research indicates that insulin resistance (IR) plays a crucial role in the development of prediabetes and T2DM (13). Dyslipidemia may impact pancreatic function and insulin sensitivity through various pathways, thereby promoting the progression of prediabetes and T2DM (14–16). The study suggests that elevated triglycerides (TG) and low high-density lipoprotein cholesterol (HDL-C) levels are significant contributors to the development of IR (17). Although the hyperinsulinemic–euglycemic clamp technique is the gold standard method for evaluating insulin resistance (18), it is not suitable for the clinical assessment of large samples due to its drawbacks of high cost, invasiveness, and long duration (19). The atherogenic index of plasma (AIP) calculated through the formula log(TG/HDL-C) (20) is regarded as a new and better indicator of dyslipidemia compared to the single lipid indicator of TG or HDL-C and is a strong marker for predicting T2DM risk (21–23). In recent years, studies have shown that AIP is strongly associated with the incidence of prediabetes or T2DM. However, these related studies are relatively limited, and the effect sizes of the results may vary due to racial differences (24–27). Therefore, we conducted a nationally representative cross-sectional study based on the China Health and Retirement Longitudinal Study (CHARLS) database. The aim was to explore the associations between AIP and the risk of prediabetes and T2DM in the Chinese population.

## Methods

### Study design and population

The CHARLS is an ongoing nationally representative longitudinal survey targeting adults aged 45 years and above. It aims to investigate the socio-demographic, economic, and health status and functioning information of the population. The baseline survey for CHARLS was conducted during, 2011–2012 in 450 communities/villages across 150 districts/counties from 28 provinces throughout the country, with subsequent follow-up surveys conducted every 2 to 3 years. Blood sample data in CHARLS were collected in, 2011 and, 2015. The National Development Institute of Peking University (IRB00001052-11015) approved the research project of CHARLS, and all participants signed an informed consent form before participating in the study.

**Figure 1:**
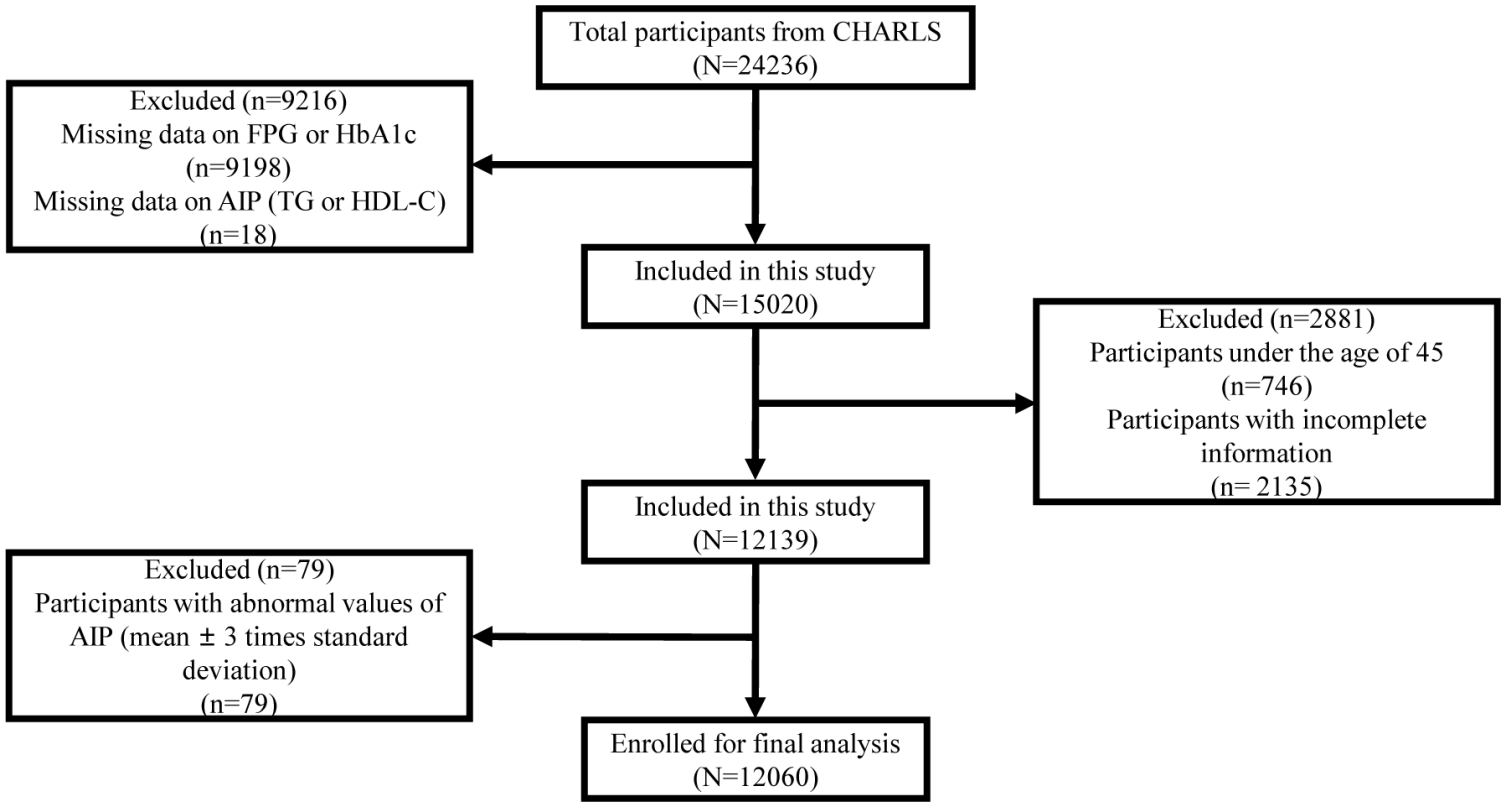
Flowchart of participant selection.

We utilized the CHARLS data (2011 and, 2015) to select eligible participants based on inclusion and exclusion criteria. We excluded individuals who were under 45 years of age; without key data on fasting plasma glucose (FPG), glycosylated hemoglobin (HbA1c), TG, and HDL-C; or with incomplete information on socio-demographic, health-related, anthropometric, and other biomarkers. We further excluded individuals with abnormal values of AIP (mean ± 3 times the standard deviation). Finally, we included 12,060 participants in this study. The exclusion process is shown in **Figure 1**.

### Data collection and definitions

Socio-demographic information (gender and age), health-related behaviors (smoking and drinking status), and medical history were collected and recorded through questionnaires by the interviewers. Anthropometric measurements including height, weight, waist circumference (WC), and blood pressure were taken by trained professionals. During the measurement of participants’ blood pressure using an electronic sphygmomanometer, three measurements of systolic blood pressure (SBP) and diastolic blood pressure (DBP) were taken, and the average of the three readings was recorded. Participants had fasting venous blood collected in the morning, including blood glucose, HbA1c, TG, total cholesterol (TC), HDL-C, low-density lipoprotein cholesterol (LDL-C), serum creatinine (Scr), blood urea nitrogen (BUN), and serum uric acid (SUA). The calculation formula for AIP was Log10(TG/HDL-C), with TG and HDL-C expressed in mg/dL. According to the American Diabetes Association guidelines (28), participants were defined as prediabetes with FPG between 100 mg/dL (5.6 mmol/L) and 125 mg/dL (6.9 mmol/L) or HbA1c between 5.7% and 6.4%. T2DM was defined as one of the following criteria: 1) FPG ≥ 126 mg/dL (7.0 mmol/L), 2) HbA1c ≥ 6.5%, 3) random plasma glucose ≥200 mg/dL (11.1 mmol/L), 4) currently receiving hypoglycemic therapy (medication or insulin injection), and 5) self-reported history of T2DM diagnosed by a physician.

### Potential covariates

We included the following covariates based on the survey questionnaire: gender, age, smoking status, alcohol consumption, SBP, DBP, body mass index (BMI), WC, TC, LDL-C, Scr, BUN, SUA, antihypertensive medications, and lipoprotein-lowering medications. BMI was categorized as follows: BMI < 18.5 kg/m^2^, 18.5 kg/m^2^ ≤ BMI < 24 kg/m^2^, and BMI ≥ 24 kg/m^2^. Abdominal obesity was defined as waist circumference ≥90 cm for men or ≥85 cm for women.

### Statistical analysis

Continuous variables are expressed as means and standard deviations, and categorical variables are described as numbers and percentages. One-way ANOVA, Kruskal–Wallis H test, or chi-square test was employed to compare the differences of variables among different quartiles of AIP. Three models were utilized in this study: Model 1, unadjusted; Model 2, adjusted for gender, age, SBP, DBP, smoking status, alcohol consumption, and BMI; Model 3, adjusted for gender, age, SBP, DBP, smoking status, alcohol consumption, BMI, WC, TC, LDL-C, Scr, BUN, SUA, antihypertensive medications, and lipoprotein-lowering medications. In this study, the associations of AIP with prediabetes and T2DM risk among participants were assessed using multivariate logistic regression analysis. In addition, a generalized additive model (GAM) was used based on smooth curve fitting to explore the non-linear relationships of AIP with the risk of prediabetes and T2DM and to observe whether there was a segmented relationship. Threshold effect analysis was performed using segmented regression models. Subsequently, multivariate logistic regression was used to perform subgroup analyses for different subgroups of gender, age, smoking status, alcohol consumption, SBP, DBP, BMI, and abdominal obesity. Interactions were also tested to assess whether these factors influenced the relationships of AIP with prediabetes and T2DM. Statistical analyses for this study were performed using EmpowerStats (http://www.empowerstats.com, X&Y Solutions, Inc., Boston, MA, USA) and the R statistical software packages (http://www.R-project.org, The R Foundation). *p* < 0.05 indicated statistical significance.

## Results

### Baseline characteristics of study participants

A total of 12,060 participants were ultimately enrolled in this study according to the inclusion and exclusion criteria. The average age of participants was 58.45 ± 9.70 years, with 5,664 men (46.97%) and 6,396 women (53.03%). The demographic and clinical characteristics of participants based on quartiles of AIP are listed in **Table 1**. All variables were statistically significant among AIP quartile groups (Q1–Q4). In comparison with the other groups, individuals in the AIP Q4 group had higher levels of SBP, DBP, BMI, WC, TC, TG, Scr, SUA, FPG, and HbA1c and lower LDL-C levels. Conversely, HDL-C was higher in the Q1 group and showed a negative association with AIP. Noteworthy, a higher proportion of individuals using antihypertensive and lipoprotein-lowering medications was observed in the AIP Q4 group compared to the lower AIP groups.

**Table 1:** Baseline characteristics of study participants according to quartiles of AIP.

Variable	Total	AIP quartiles				*p*-Value
		Q1 (<0.14)	Q2 (0.14–0.33)	Q3 (0.33–0.56)	Q4 (≥0.56)	
Number	12,060	3,014	3,016	3,015	3,015	
Gender, n (%)						<0.001
Male	5,664 (46.97%)	1,534 (50.90%)	1,413 (46.85%)	1,312 (43.52%)	1,405 (46.60%)	
Female	6,396 (53.03%)	1,480 (49.10%)	1,603 (53.15%)	1,703 (56.48%)	1,610 (53.40%)	
Age (years)	58.45 ± 9.70	59.14 ± 10.01	58.53 ± 9.86	58.46 ± 9.68	57.65 ± 9.19	<0.001
SBP (mmHg)	129.42 ± 20.98	126.76 ± 20.71	127.98 ± 20.76	130.64 ± 21.19	132.32 ± 20.84	<0.001
DBP (mmHg)	75.76 ± 11.97	73.57 ± 11.78	74.82 ± 11.59	76.54 ± 12.04	78.10 ± 12.00	<0.001
BMI (kg/m^2^)	23.66 ± 3.87	21.93 ± 3.23	23.13 ± 3.73	24.23 ± 3.80	25.37 ± 3.83	<0.001
WC (cm)	84.59 ± 12.65	79.78 ± 10.82	83.05 ± 12.22	86.17 ± 12.36	89.37 ± 13.03	<0.001
Smoking status, n (%)						<0.001
Never smoker	7,302 (60.55%)	1,737 (57.63%)	1,835 (60.84%)	1,907 (63.25%)	1,823 (60.46%)	
Ever smoker	1,118 (9.27%)	270 (8.96%)	270 (8.95%)	265 (8.79%)	313 (10.38%)	
Current smoker	3,640 (30.18%)	1,007 (33.41%)	911 (30.21%)	843 (27.96%)	879 (29.15%)	
Alcohol consumption, n (%)						<0.001
Never drinker	6,906 (57.26%)	1,593 (52.85%)	1,734 (57.49%)	1,795 (59.54%)	1,784 (59.17%)	
Ever drinker	1,021 (8.47%)	209 (6.93%)	269 (8.92%)	292 (9.68%)	251 (8.33%)	
Current drinker	4,133 (34.27%)	1,212 (40.21%)	1,013 (33.59%)	928 (30.78%)	980 (32.50%)	
TC (mg/dL)	190.85 ± 37.76	185.92 ± 34.52	187.04 ± 36.50	192.05 ± 37.32	198.38 ± 41.11	<0.001
TG (mg/dL)	131.62 ± 83.49	63.87 ± 15.01	93.14 ± 18.48	130.02 ± 26.97	239.43 ± 94.31	<0.001
HDL-C (mg/dL)	51.29 ± 14.14	65.63 ± 13.51	53.72 ± 9.76	46.97 ± 8.43	38.84 ± 8.11	<0.001
LDL-C (mg/dL)	113.54 ± 33.63	108.86 ± 30.21	115.61 ± 32.43	119.94 ± 33.87	109.76 ± 36.51	<0.001
Scr (mg/dL)	0.79 ± 0.27	0.78 ± 0.30	0.78 ± 0.21	0.79 ± 0.29	0.80 ± 0.25	<0.001
BUN (mg/dL)	15.55 ± 4.61	16.36 ± 4.97	15.60 ± 4.36	15.18 ± 4.62	15.04 ± 4.35	<0.001
SUA (mg/dL)	4.57 ± 1.31	4.29 ± 1.17	4.44 ± 1.26	4.60 ± 1.30	4.94 ± 1.40	<0.001
FPG (mg/dL)	108.01 ± 35.68	101.01 ± 24.32	103.41 ± 29.42	107.92 ± 35.04	119.69 ± 46.91	<0.001
HbA1c (%)	5.42 ± 0.89	5.26 ± 0.70	5.37 ± 0.79	5.44 ± 0.87	5.63 ± 1.09	<0.001
Antihypertensive drugs	2,358 (19.55%)	368 (12.21%)	485 (16.08%)	661 (21.92%)	844 (27.99%)	<0.001
Lipoprotein-lowering drugs	641 (5.32%)	84 (2.79%)	115 (3.81%)	167 (5.54%)	275 (9.12%)	<0.001

Data are presented as mean ± standard deviation or number (%).

AIP, atherogenic index of plasma; SBP, systolic blood pressure; DBP, diastolic blood pressure; BMI, body mass index; WC, waist circumference; TC, total cholesterol; TG, triglyceride; HDL-C, high-density lipoprotein cholesterol; LDL-C, low-density lipoprotein cholesterol; Scr, serum creatinine; BUN, blood urea nitrogen; SUA, serum uric acid; FPG, fasting plasma glucose; HbA1c, hemoglobin A1c.

### Multivariate regression analysis

**Table 2** presents the odds ratios (ORs) and 95% confidence intervals (CIs) for the associations between AIP and the risk of prediabetes and T2DM in different models. In the unadjusted model (Model 1), our results unveiled a positive association between AIP and prediabetes risk (OR = 2.57, 95% CI: 2.23–2.95). After adjusting for the potential confounders (Model 2: OR = 2.06, 95% CI: 1.77–2.40; Model 3: OR = 1.75, 95% CI: 1.49–2.06), the association between AIP and prediabetes remained significant. Similarly, regardless of whether the potential confounders were adjusted, a significant positive association between AIP and T2DM risk was also observed in different models (Model 1: OR = 5.01, 95% CI: 4.25–5.90; Model 2: OR = 3.78, 95% CI: 3.17–4.52; Model 3: OR = 2.91, 95% CI: 2.38–3.57). When AIP was considered as a categorical variable, in the fully adjusted Model 3, compared to the lowest quartile of AIP (Q1 group), the adjusted ORs for prediabetes in the Q2, Q3, and Q4 groups were 1.07 (95% CI: 0.95–1.20), 1.25 (95% CI: 1.11–1.41), and 1.52 (95% CI: 1.33–1.74), respectively. For T2DM, the adjusted ORs in the Q2, Q3, and Q4 groups were 1.25 (95% CI: 1.05–1.49), 1.60 (95% CI: 1.35–1.90), and 2.28 (95% CI: 1.92–2.71), respectively. The results indicated a progressive increase in the risk of developing prediabetes and T2DM with elevated AIP levels.

**Table 2:** Multivariate regression analysis of the association between AIP and prediabetes and T2DM.

Variable	Model 1		Model 2		Model 3	
	OR (95% CI)	*p*-Value	OR (95% CI)	*p*-Value	OR (95% CI)	*p*-Value
Prediabetes						
AIP	2.57 (2.23, 2.95)	<0.0001	2.06 (1.77, 2.40)	<0.0001	1.75 (1.49, 2.06)	<0.0001
Q1 (<0.14)	Reference		Reference		Reference	
Q2 (0.14–0.33)	1.10 (0.99, 1.23)	0.0795	1.04 (0.93, 1.16)	0.5463	1.07 (0.95, 1.20)	0.2484
Q3 (0.33–0.56)	1.43 (1.28, 1.59)	<0.0001	1.24 (1.11, 1.40)	0.0002	1.25 (1.11, 1.41)	0.0003
Q4 (≥0.56)	2.06 (1.83, 2.32)	<0.0001	1.73 (1.52, 1.96)	<0.0001	1.52 (1.33, 1.74)	<0.0001
*p* for trend	<0.0001		<0.0001		<0.0001	
T2DM						
AIP	5.01 (4.25, 5.90)	<0.0001	3.78 (3.17, 4.52)	<0.0001	2.91 (2.38, 3.57)	<0.0001
Q1 (<0.14)	Reference		Reference		Reference	
Q2 (0.14–0.33)	1.31 (1.11, 1.55)	0.0018	1.18 (0.99, 1.40)	0.0588	1.25 (1.05, 1.49)	0.0138
Q3 (0.33–0.56)	1.88 (1.60, 2.20)	<0.0001	1.53 (1.29, 1.80)	<0.0001	1.60 (1.35, 1.90)	<0.0001
Q4 (≥0.56)	3.46 (2.97, 4.03)	<0.0001	2.63 (2.24, 3.09)	<0.0001	2.28 (1.92, 2.71)	<0.0001
*p* for trend	<0.0001		<0.0001		<0.0001	

Model 1 adjusted for none. Model 2 adjusted for gender, age, SBP, DBP, smoking status, alcohol consumption, and BMI. Model 3 adjusted for Model 2 + WC, TC, LDL-C, Scr, BUN, SUA, antihypertensive drugs, and lipoprotein-lowering drugs. AIP as a continuous variable and quartiles variable (Q1, Q2, Q3, and Q4).

AIP, atherogenic index of plasma; OR, odds ratio; CI, confidence interval; T2DM, type 2 diabetes mellitus; SBP, systolic blood pressure; DBP, diastolic blood pressure; BMI, body mass index; WC, waist circumference; TC, total cholesterol; LDL-C, low-density lipoprotein cholesterol; Scr, serum creatinine; BUN, blood urea nitrogen; SUA, serum uric acid.

### Subgroup analysis

**Figure 2:**
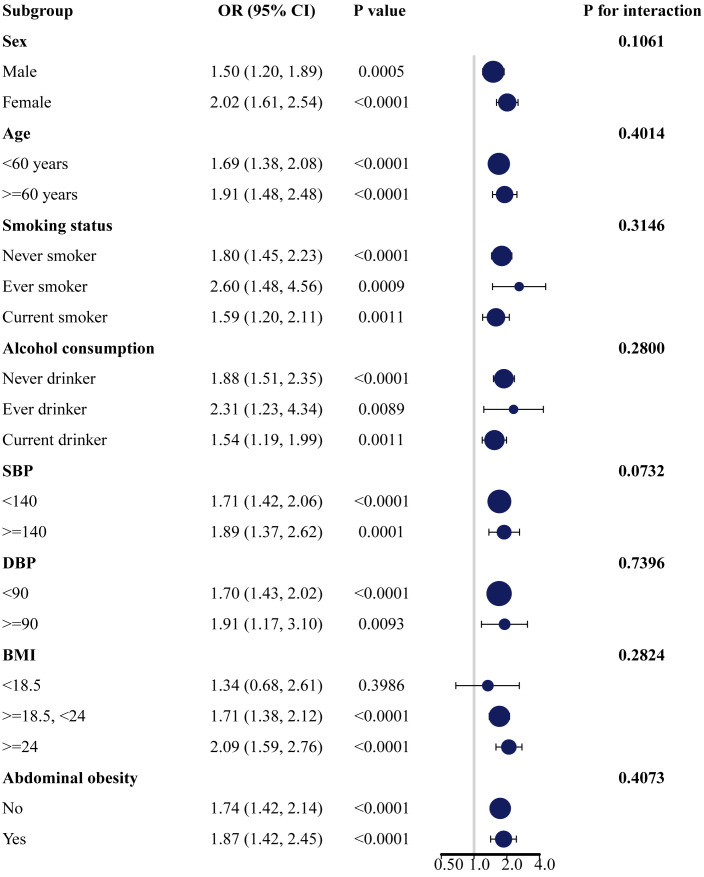
Subgroup analysis of the association between AIP and the risk of prediabetes. AIP, atherogenic index of plasma.

**Figure 3:**
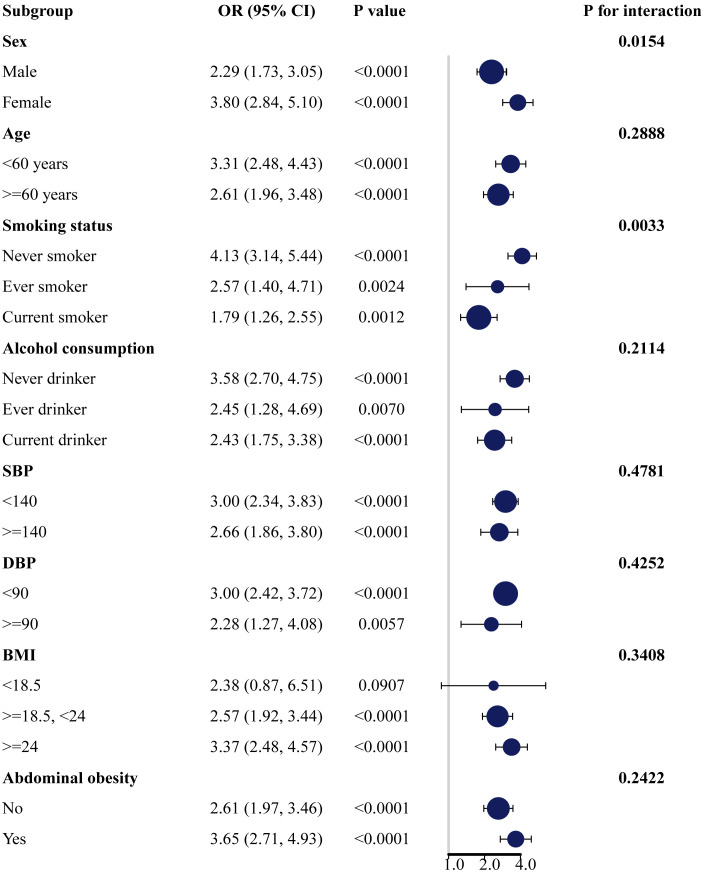
Subgroup analysis of the association between AIP and the risk of T2DM. AIP, atherogenic index of plasma; T2DM, type 2 diabetes mellitus.

Subgroup analyses were conducted based on gender, age, smoking status, alcohol consumption, SBP, DBP, BMI, and abdominal obesity to explore the associations of AIP with the risk of prediabetes and T2DM in different subgroups. The results revealed significant positive associations between AIP and the risk of prediabetes (**Supplementary Table S1**; **Figure 2**) and T2DM (**Supplementary Table S2**; **Figure 3**) across different subgroups. Additionally, women had a higher risk of developing prediabetes (OR = 2.02, 95% CI: 1.61–2.54) or T2DM (OR = 3.80, 95% CI: 2.84–5.10) compared to men. A stronger association between AIP and the risk of T2DM was also observed in non-smokers and non-drinkers. In this study, gender (*p* for interaction = 0.0154) and smoking (*p* for interaction = 0.0033) were identified as significant interacting factors influencing the relationship between AIP and T2DM. However, no significant interaction was observed between subgroups and the association of AIP with the risk of prediabetes.

### Non-linear relationship

**Figure 4:**
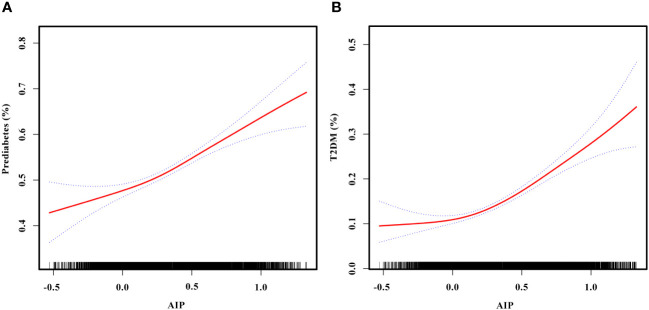
**(A)** Smooth curve fitting to evaluate the non-linear relationship between AIP and the risk of prediabetes. The red solid line represents the probability of prediabetes occurrence, and the blue dotted line represents the 95% CI curve. **(B)** Smooth curve fitting to evaluate the non-linear relationship between AIP and the risk of T2DM. The red solid line represents the probability of T2DM occurrence, and the blue dotted line represents the 95% CI curve. AIP, atherogenic index of plasma; T2DM, type 2 diabetes mellitus.

The non-linear relationships of AIP with the risk of prediabetes and T2DM were analyzed through a GAM and smooth curve fitting. After adjusting for gender, age, SBP, DBP, smoking status, alcohol consumption, BMI, WC, TC, LDL-C, Scr, BUN, SUA, antihypertensive medications, and lipoprotein-lowering medications, the results revealed non-linear relationships of AIP with the risk of prediabetes (**Figure 4A**) and T2DM (**Figure 4B**). The inflection points for investigating the relationships of AIP with the risk of prediabetes and T2DM were identified by threshold effect analysis. **Table 3** shows that the inflection point for AIP in prediabetic patients was 0.29. When AIP > 0.29, there was a positive association between AIP and the risk of prediabetes (OR = 2.24, 95% CI: 1.67–3.00, *p* < 0.0001). However, when AIP < 0.29, AIP was not associated with the risk of prediabetes (OR = 1.28, 95% CI: 0.91–1.81, *p* = 0.1597). The AIP inflection point for diabetic patients was −0.04 as shown in **Table 4**. Similarly, When AIP > −0.04, AIP showed a positive association with the risk of T2DM (OR = 3.33, 95% CI: 2.67–4.16, *p* < 0.0001). However, when AIP < −0.04, AIP was not associated with the risk of T2DM (OR = 0.30, 95% CI: 0.06–1.39, *p* = 0.1235).

**Table 3:** Threshold effect analysis of AIP on prediabetes.

AIP	Adjusted OR (95% CI), *p*-value
Model 1	
Fitting by the standard linear model	1.75 (1.49, 2.06), < 0.0001
Model 2	
Inflection point	0.29
<0.29	1.28 (0.91, 1.81), 0.1597
>0.29	2.24 (1.67, 3.00), < 0.0001
Log likelihood ratio	0.003

Adjusted for gender, age, SBP, DBP, smoking status, alcohol consumption, BMI, WC, TC, LDL-C, Scr, BUN, SUA, antihypertensive drugs, and lipoprotein-lowering drugs.

AIP, atherogenic index of plasma; OR, odds ratio; CI, confidence interval; SBP, systolic blood pressure; DBP, diastolic blood pressure; BMI, body mass index; WC, waist circumference; TC, total cholesterol; LDL-C, low-density lipoprotein cholesterol; Scr, serum creatinine; BUN, blood urea nitrogen; SUA, serum uric acid.

**Table 4:** Threshold effect analysis of AIP on T2DM.

AIP	Adjusted OR (95% CI), *p*-value
Model 1	
Fitting by the standard linear model	2.91 (2.38, 3.57), < 0.0001
Model 2	
Inflection point	−0.04
<−0.04	0.30 (0.06, 1.39), 0.1235
>−0.04	3.33 (2.67, 4.16), < 0.0001
Log likelihood ratio	0.005

Adjusted for gender, age, SBP, DBP, smoking status, alcohol consumption, BMI, WC, TC, LDL-C, Scr, BUN, SUA, antihypertensive drugs, and lipoprotein-lowering drugs.

AIP, atherogenic index of plasma; OR, odds ratio; CI, confidence interval; T2DM, type 2 diabetes mellitus; SBP, systolic blood pressure; DBP, diastolic blood pressure; BMI, body mass index; WC, waist circumference; TC, total cholesterol; LDL-C, low-density lipoprotein cholesterol; Scr, serum creatinine; BUN, blood urea nitrogen; SUA, serum uric acid.

## Discussion

In this cross-sectional study, our results indicated positive associations between AIP and the risk of prediabetes and T2DM in the Chinese population aged 45 years and above. In addition, higher AIP was significantly associated with prediabetes and T2DM risk in both male and female populations. Notably, the association was stronger in women compared to men. A stronger association between AIP and T2DM risk was also observed in non-smokers and non-drinkers.

A cross-sectional survey involving 9,245 participants in the United States revealed that AIP was associated with increased risk of IR (OR = 1.29, 95% CI: 1.26–1.32) and T2DM (OR = 1.18, 95% CI: 1.15–1.22). This suggested that AIP had the potential to serve as a monitoring indicator for IR and T2DM (26). In a study conducted on a rural population in Bangladesh, the results showed that high levels of TG and low levels of HDL-C were strongly associated with prediabetes and T2DM (29). Another study evaluating the association between AIP and its longitudinal effect on T2DM in middle-aged and older Chinese reported that individuals with higher baseline AIP were more likely to develop T2DM compared with those with lower baseline AIP (24). Our results were consistent with previous studies showing that the risk of prediabetes and T2DM increases with elevated AIP.

Studies have shown that IR is a key pathological driver in the development of prediabetes and T2DM (30). Although the hyperinsulinemic–euglycemic clamp test is the gold standard method for evaluating insulin resistance, its limitations including its high cost, invasiveness, and time-consuming nature, make it unsuitable for large-scale clinical studies (18, 19). Previous studies have confirmed that abnormal lipid metabolism is an independent risk factor for prediabetes and T2DM (31). Dyslipidemia may impact pancreatic function and insulin sensitivity through various pathways, promoting the progression of prediabetes and T2DM (14–16). Studies indicate that high TG and low HDL-C levels play a crucial role in the development of IR (17). Higher concentrations of TG increase free fatty acids, decrease insulin sensitivity, and contribute to insulin resistance (32, 33). Lower levels of HDL-C reduce cholesterol efflux and increase cholesterol accumulation in pancreatic β-cells, affecting pancreatic function (34, 35). The AIP, composed of TG and HDL-C, is a cost-effective and widely used marker in routine blood tests. Compared with single lipid indicators such as TG, TC, HDL-C, and LDL-C, the AIP is considered a new and better indicator of dyslipidemia and has significant value in the prediction of T2DM (21–23). In addition, high levels of AIP are also closely associated with the development of various macrovascular and microvascular complications of T2DM, including coronary artery disease, nephropathy, retinopathy, and metabolic syndrome (25, 36–41).

In **Table 1**, LDL-C was lower in the AIP Q4 group than in the other groups, and we have observed similar results in previous studies (42, 43). Previous studies have shown that AIP is not associated with LDL-C (37, 44). Elevated AIP is significantly associated with a higher risk of T2DM, and the Q4 group with higher AIP had relatively more patients with T2DM. Although it is widely recognized that LDL-C is a key factor in cardiovascular disease, whether high or low LDL-C levels influence the development of diabetes remains controversial. Previous studies have indicated that LDL-C is mostly normal in T2DM patients with abnormal lipid metabolism (45), that elevated LDL-C does not lead to a significant increase in T2DM, and genetic researches indicate that reduced LDL-C may be a protective factor for T2DM (31, 46). Therefore, it may cause the phenomenon that the Q4 group with higher AIP (more diabetic patients) had lower LDL-C than the other groups. When assessing the association between AIP and the risk of prediabetes in the three different models in **Table 2**, we found that there was no correlation between lower levels of AIP (AIP Q2) and the risk of prediabetes. **Table 3** shows that the inflection point for AIP in prediabetic patients was 0.29, and AIP was not associated with prediabetes risk when AIP < 0.29. The range of AIP Q2 was mostly in the left portion of the inflection point of 0.29, which was consistent with the result that AIP Q2 was not associated with prediabetes risk in **Table 2**. The possible reason is that when AIP is low, the levels of the risk factors for prediabetes, such as BMI, FPG, and TC are also low, leading to a weak impact on prediabetes. As AIP rises (Q3 and Q4), AIP begins to be positively associated with the risk of prediabetes.

The associations of AIP with prediabetes and T2DM risk may vary based on factors such as gender, age, smoking status, and alcohol consumption. In subgroup analyses, we observed positive associations between AIP and the risk of prediabetes and T2DM in all subgroup variables. In addition, the association of AIP with T2DM risk was more pronounced in women, non-smokers, and non-drinkers. Our study showed that higher AIP was significantly positively associated with the risk of T2DM in both male and female populations, with a stronger association observed in women. This result is consistent with a cross-sectional study of the non-linear relationship of AIP with T2DM in the general US population and a case-control study of the association between TG/HDL-C and the incidence of T2DM in Singapore Chinese men and women (26, 47). In a longitudinal study assessing the association between AIP and T2DM risk in the Taiwanese population, no gender difference was found, and a higher risk of T2DM was observed only in participants aged 40-64 years (25). This differs from our results, which found that participants included in the study had a higher risk of developing T2DM in all different age groups (*p* < 0.0001). This may be attributed to differences in the characteristics of the study populations. A study involving U.S. adults aged 18 years and older explored gender differences in the impact of AIP on prediabetes and diabetes. The results indicated that with each unit increase in AIP, the prevalence of prediabetes and diabetes in female participants increased by 4.96 times (OR = 4.96, 95% CI: 2.68-9.18). However, there was no significant association between AIP and the prevalence of prediabetes or diabetes in male participants (48). This gender-specific impact may be related to the physiological cycle in women influenced by hormone levels after menopause, leading to lipid metabolism disturbances and the onset of cardiovascular diseases, T2DM, and metabolic syndrome (49). Previous studies have shown that the association between dyslipidemia and T2DM seems to be stronger in smoking and alcohol-drinking populations (50). However, our study revealed that the association between AIP and T2DM was stronger in non-smokers and non-drinkers compared to smokers and drinkers. In a study on Chinese patients with coronary heart disease, the associations between TG/HDL-C and other non-traditional lipid parameters with the risk of prediabetes and T2DM were stronger in non-smokers and non-drinkers (51). This result is consistent with our findings. The reasons for these differences may be influenced by factors such as gender, race, and sample size. Therefore, further research is needed to explore the impact of these variables on the relationships between AIP and the risk of prediabetes and T2DM.

After adjusting for gender, age, SBP, DBP, smoking status, alcohol consumption, BMI, WC, TC, LDL-C, Scr, BUN, SUA, antihypertensive medications, and lipoprotein-lowering medications, our study revealed non-linear relationships of AIP with prediabetes and T2DM risk. Threshold effect analysis showed that the associations of AIP with the risk of prediabetes and T2DM differed on either side of the inflection points. In individuals with prediabetes, the inflection point for AIP was 0.29. When AIP > 0.29, AIP was positively associated with the risk of prediabetes (OR = 2.24, 95% CI: 1.67–3.00, *p* < 0.0001). However, when AIP < 0.29, AIP was not associated with the risk of prediabetes (OR = 1.28, 95% CI: 0.91–1.81, *p* = 0.1597). In patients with T2DM, the inflection point for AIP was −0.04. Similarly, when AIP > −0.04, AIP was positively associated with the risk of T2DM (OR = 3.33, 95% CI: 2.67–4.16, *p* < 0.0001). However, when AIP < −0.04, AIP was not associated with the risk of T2DM (OR = 0.30, 95% CI: 0.06–1.39, *p* = 0.1235). Regarding the difference in AIP thresholds between prediabetes and T2DM in this study, we speculate that it may be related to the different sensitivities to AIP caused by the different pathological mechanisms and metabolic states of prediabetes and T2DM. Prediabetes represents an early stage of T2DM, characterized by a relatively mild condition that may have strong metabolic regulatory capacity. This could imply that the association between AIP and the risk of prediabetes requires higher AIP levels to achieve significance. In contrast, at the T2DM stage, the condition tends to be more severe and may increase sensitivity to AIP, making the association between AIP and the risk of T2DM significant at lower AIP levels. It is worth noting that these speculations are based on existing data, and further research is needed for exploration in the future. The AIP can serve as a potential early warning indicator, predicting the risk of developing prediabetes and T2DM. This may contribute to alerting individuals to adopt healthy dietary habits, increase physical activity, and have regular medical checkups, thereby reducing the risk of progressing to prediabetes and T2DM.

This study has several major strengths. First, it benefits from a large sample size, with data sourced from the CHARLS database. Trained professionals conducted the collection of comprehensive data, including demographic information, health behaviors, anthropometric measurements, and laboratory tests, enhancing the reliability of the study results. Second, subgroup analyses were conducted based on gender, age, smoking status, alcohol consumption, SBP, DBP, BMI, and abdominal obesity. These analyses aimed to assess whether these factors influence the relationships between AIP and the risk of prediabetes and T2DM, validating the stability of the models.

However, this study also has certain limitations. First, given the adoption of a cross-sectional study design, our findings can only uncover the associations between AIP and the risk of prediabetes and T2DM. To establish the actual causal relationship, further prospective studies are essential. Second, despite controlling and adjusting for some potential confounding factors in our study, the influence of unknown factors cannot be completely excluded. Finally, our study results are specific to the Chinese population, and the feasibility of generalization to other populations requires further exploration. We encourage future research to consider potential confounding factors and conduct more comprehensive investigations on a broader population.

## Conclusions

In conclusion, our study showed positive non-linear associations between AIP and the risk of prediabetes and T2DM. The AIP shows good potential in predicting the risk of prediabetes and T2DM among the middle-aged and elderly Chinese population, holding practical significance for the prevention and management of prediabetes and T2DM. In the future, the AIP may be expected to be a valuable monitoring tool for the risk of prediabetes and T2DM, but more studies are needed for in-depth analysis and exploration.

## Data availability statement

The raw data supporting the conclusions of this article will be made available by the authors, without undue reservation.

## Ethics statement

The studies involving humans were approved by Ethical Review Committee of Peking University (IRB00001052-11015). The studies were conducted in accordance with the local legislation and institutional requirements. The human samples used in this study were acquired from https://charls.charlsdata.com/pages/data/111/zh-cn.html. Written informed consent for participation was not required from the participants or the participants’ legal guardians/next of kin in accordance with the national legislation and institutional requirements.

## Author contributions

LJ: Writing – review & editing, Writing – original draft, Software, Resources, Project administration, Methodology, Formal analysis, Data curation, Conceptualization. LL: Writing – review & editing, Formal analysis, Data curation. ZX: Writing – review & editing, Formal analysis, Data curation. YT: Writing – review & editing, Formal analysis, Data curation. YZ: Writing – review & editing, Formal analysis, Data curation. XF: Writing – review & editing, Formal analysis. DL: Writing – review & editing, Conceptualization. QW: Writing – review & editing, Conceptualization.
