# Supplementary material for: Non-linear associations of atherogenic index of plasma with prediabetes and type 2 diabetes mellitus among Chinese adults aged 45 years and above: a cross-sectional study from CHARLS
Source: Front Endocrinol (Lausanne). 2024 Apr 2;15:1360874. doi: 10.3389/fendo.2024.1360874 (PMC11018972; doi:10.3389/fendo.2024.1360874)
Supplement: Supplementary file 1 [file Table_1.docx]

Supplementary Material

**Supplementary Table S1. Subgroup analysis of the associations between AIP and prediabetes.**

| AIP | Total | OR(95%CI) | | | | *P* for trend | *P* for interaction |
| --- | --- | --- | --- | --- | --- | --- | --- |
|  |  | Q1(<0.14) | Q2(0.14–0.33) | Q3(0.33–0.56) | Q4(≥ 0.56) |  |  |
| Gender |  |  |  |  |  |  | 0.1061 |
| Male | 1.50 (1.20, 1.89) | Reference | 1.10 (0.93, 1.29) | 1.25 (1.05, 1.50) | 1.40 (1.15, 1.70) | 0.0003 |  |
| Female | 2.02 (1.61, 2.54) | Reference | 1.03 (0.88, 1.21) | 1.24 (1.06, 1.47) | 1.64 (1.36, 1.97) | <0.0001 |  |
| Age (years) |  |  |  |  |  |  | 0.4014 |
| <60 | 1.69 (1.38, 2.08) | Reference | 1.03 (0.89, 1.20) | 1.17 (1.00, 1.37) | 1.50 (1.26, 1.78) | <0.0001 |  |
| ≥60 | 1.91 (1.48, 2.48) | Reference | 1.10 (0.92, 1.32) | 1.37 (1.14, 1.65) | 1.61 (1.30, 2.00) | <0.0001 |  |
| Smoking status |  |  |  |  |  |  | 0.3146 |
| Never smoker | 1.80 (1.45, 2.23) | Reference | 0.99 (0.86, 1.15) | 1.19 (1.02, 1.39) | 1.52 (1.28, 1.81) | <0.0001 |  |
| Ever smoker | 2.60 (1.48, 4.56) | Reference | 1.03 (0.68, 1.56) | 1.42 (0.92, 2.19) | 2.33 (1.42, 3.81) | 0.0004 |  |
| Current smoker | 1.59 (1.20, 2.11) | Reference | 1.27 (1.04, 1.56) | 1.39 (1.12, 1.73) | 1.41 (1.11, 1.79) | 0.0022 |  |
| Alcohol consumption |  |  |  |  |  |  | 0.2800 |
| Never drinker | 1.88 (1.51, 2.35) | Reference | 1.06 (0.91, 1.24) | 1.29 (1.10, 1.51) | 1.55 (1.30, 1.86) | <0.0001 |  |
| Ever drinker | 2.31 (1.23, 4.34) | Reference | 1.01 (0.66, 1.56) | 1.23 (0.79, 1.92) | 2.18 (1.28, 3.70) | 0.0035 |  |
| Current drinker | 1.54 (1.19, 1.99) | Reference | 1.10 (0.91, 1.33) | 1.20 (0.98, 1.47) | 1.40 (1.12, 1.75) | 0.0025 |  |
| SBP (mmHg) |  |  |  |  |  |  | 0.0732 |
| <140 | 1.71 (1.42, 2.06) | Reference | 1.10 (0.97, 1.25) | 1.17 (1.02, 1.34) | 1.51 (1.30, 1.77) | <0.0001 |  |
| ≥140 | 1.89 (1.37, 2.62) | Reference | 0.97 (0.76, 1.23) | 1.53 (1.19, 1.95) | 1.58 (1.21, 2.07) | <0.0001 |  |
| DBP (mmHg) |  |  |  |  |  |  | 0.7396 |
| <90 | 1.70 (1.43, 2.02) | Reference | 1.04 (0.93, 1.18) | 1.21 (1.07, 1.38) | 1.49 (1.29, 1.71) | <0.0001 |  |
| ≥90 | 1.91 (1.17, 3.10) | Reference | 1.35 (0.92, 1.99) | 1.55 (1.06, 2.27) | 1.84 (1.22, 2.76) | 0.0030 |  |
| BMI (kg/m^2^) |  |  |  |  |  |  | 0.2824 |
| <18.5 | 1.34 (0.68, 2.61) | Reference | 0.81 (0.54, 1.21) | 1.40 (0.86, 2.29) | 1.36 (0.66, 2.82) | 0.2502 |  |
| ≥18.5, <24 | 1.71 (1.38, 2.12) | Reference | 1.07 (0.93, 1.24) | 1.25 (1.06, 1.46) | 1.51 (1.26, 1.81) | <0.0001 |  |
| ≥24 | 2.09 (1.59, 2.76) | Reference | 1.16 (0.93, 1.44) | 1.30 (1.05, 1.61) | 1.66 (1.33, 2.09) | <0.0001 |  |
| Abdominal obesity |  |  |  |  |  |  | 0.4073 |
| No | 1.74 (1.42, 2.14) | Reference | 1.02 (0.89, 1.17) | 1.27 (1.10, 1.48) | 1.58 (1.32, 1.88) | <0.0001 |  |
| Yes | 1.87 (1.42, 2.45) | Reference | 1.23 (0.98, 1.53) | 1.30 (1.05, 1.61) | 1.55 (1.24, 1.95) | <0.0001 |  |

The adjustment factors included gender, age, SBP, DBP, smoking status, alcohol consumption, BMI, WC, TC, LDL-C, Scr, BUN, SUA, antihypertensive drugs, lipoprotein-lowering drugs.

AIP as a continuous variable and quartiles variable (Q1, Q2, Q3, and Q4); AIP, atherogenic index of plasma; OR, odds ratio; CI, confidence interval.

**Supplementary Table S2. Subgroup analysis of the associations between AIP and T2DM.**

| AIP | Total | OR(95%CI) | | | | *P* for trend | *P* for interaction |
| --- | --- | --- | --- | --- | --- | --- | --- |
|  |  | Q1(<0.14) | Q2(0.14–0.33) | Q3(0.33–0.56) | Q4(≥ 0.56) |  |  |
| Gender |  |  |  |  |  |  | 0.0154 |
| Male | 2.29 (1.73, 3.05) | Reference | 1.08 (0.84, 1.38) | 1.26 (0.99, 1.62) | 1.85 (1.44, 2.36) | <0.0001 |  |
| Female | 3.80 (2.84, 5.10) | Reference | 1.46 (1.13, 1.89) | 2.01 (1.57, 2.56) | 2.82 (2.20, 3.62) | <0.0001 |  |
| Age (years) |  |  |  |  |  |  | 0.2888 |
| <60 | 3.31 (2.48, 4.43) | Reference | 1.42 (1.10, 1.84) | 1.79 (1.39, 2.30) | 2.49 (1.94, 3.20) | <0.0001 |  |
| ≥60 | 2.61 (1.96, 3.48) | Reference | 1.08 (0.85, 1.38) | 1.43 (1.13, 1.82) | 2.12 (1.67, 2.69) | <0.0001 |  |
| Smoking status |  |  |  |  |  |  | 0.0033 |
| Never smoker | 4.13 (3.14, 5.44) | Reference | 1.51 (1.19, 1.92) | 2.15 (1.71, 2.71) | 3.05 (2.41, 3.85) | <0.0001 |  |
| Ever smoker | 2.57 (1.40, 4.71) | Reference | 1.01 (0.59, 1.71) | 1.07 (0.62, 1.85) | 2.05 (1.22, 3.45) | 0.0038 |  |
| Current smoker | 1.79 (1.26, 2.55) | Reference | 0.98 (0.72, 1.33) | 1.07 (0.78, 1.46) | 1.46 (1.08, 1.98) | 0.0097 |  |
| Alcohol consumption |  |  |  |  |  |  | 0.2114 |
| Never drinker | 3.58 (2.70, 4.75) | Reference | 1.36 (1.06, 1.73) | 1.72 (1.36, 2.17) | 2.58 (2.04, 3.27) | <0.0001 |  |
| Ever drinker | 2.45 (1.28, 4.69) | Reference | 0.91 (0.53, 1.57) | 1.03 (0.60, 1.76) | 1.75 (1.01, 3.03) | 0.0191 |  |
| Current drinker | 2.43 (1.75, 3.38) | Reference | 1.25 (0.93, 1.68) | 1.75 (1.31, 2.33) | 2.11 (1.58, 2.82) | <0.0001 |  |
| SBP (mmHg) |  |  |  |  |  |  | 0.4781 |
| <140 | 3.00 (2.34, 3.83) | Reference | 1.27 (1.03, 1.57) | 1.58 (1.29, 1.95) | 2.32 (1.88, 2.85) | <0.0001 |  |
| ≥140 | 2.66 (1.86, 3.80) | Reference | 1.14 (0.83, 1.58) | 1.60 (1.18, 2.17) | 2.13 (1.57, 2.89) | <0.0001 |  |
| DBP (mmHg) |  |  |  |  |  |  | 0.4252 |
| <90 | 3.00 (2.42, 3.72) | Reference | 1.22 (1.01, 1.47) | 1.65 (1.37, 1.97) | 2.27 (1.90, 2.73) | <0.0001 |  |
| ≥90 | 2.28 (1.27, 4.08) | Reference | 1.52 (0.86, 2.68) | 1.28 (0.74, 2.22) | 2.30 (1.34, 3.92) | 0.0020 |  |
| BMI (kg/m^2^) |  |  |  |  |  |  | 0.3408 |
| <18.5 | 2.38 (0.87, 6.51) | Reference | 0.94 (0.49, 1.78) | 1.47 (0.70, 3.10) | 1.94 (0.70, 5.36) | 0.1703 |  |
| ≥18.5, <24 | 2.57 (1.92, 3.44) | Reference | 1.18 (0.93, 1.49) | 1.34 (1.05, 1.70) | 2.20 (1.73, 2.80) | <0.0001 |  |
| ≥24 | 3.37 (2.48, 4.57) | Reference | 1.49 (1.09, 2.03) | 2.01 (1.50, 2.70) | 2.63 (1.96, 3.53) | <0.0001 |  |
| Abdominal obesity |  |  |  |  |  |  | 0.2422 |
| No | 2.61 (1.97, 3.46) | Reference | 1.19 (0.95, 1.48) | 1.43 (1.14, 1.80) | 2.23 (1.76, 2.82) | <0.0001 |  |
| Yes | 3.65 (2.71, 4.93) | Reference | 1.46 (1.07, 1.99) | 1.96 (1.47, 2.62) | 2.74 (2.06, 3.65) | <0.0001 |  |

The adjustment factors included gender, age, SBP, DBP, smoking status, alcohol consumption, BMI, WC, TC, LDL-C, Scr, BUN, SUA, antihypertensive drugs, lipoprotein-lowering drugs.

AIP as a continuous variable and quartiles variable (Q1, Q2, Q3, and Q4); AIP, atherogenic index of plasma; OR, odds ratio; CI, confidence interval.
